# Boxing the Ears

**Published:** 1874-10

**Authors:** 


### BOXING THE EARS.

The drum of the ear is as thin as thin paper, and is stretched like a curtain between the air outside and that within; and thus having nothing to support it, and being extremely delicate, a slap with the hand on the side of the face, made with the force which sudden and violent anger gives it, has in multitudes of cases ruptured this delicate membrane, resulting in the affliction of deafness for life. As the right hand is almost always used, it is the left ear which is stricken; this aids in accounting for the fact that the left ear is more frequently affected with deafness than the right.

